# Glutamine Supplementation Stimulates Protein-Synthetic and Inhibits Protein-Degradative Signaling Pathways in Skeletal Muscle of Diabetic Rats

**DOI:** 10.1371/journal.pone.0050390

**Published:** 2012-12-11

**Authors:** Adriana C. Lambertucci, Rafael H. Lambertucci, Sandro M. Hirabara, Rui Curi, Anselmo S. Moriscot, Tatiana C. Alba-Loureiro, Lucas Guimarães-Ferreira, Adriana C. Levada-Pires, Diogo A. A. Vasconcelos, Donald F. Sellitti, Tania C. Pithon-Curi

**Affiliations:** 1 Institute of Physical Activity Sciences and Sports, Post-Graduate Program in Human Movement Sciences, Cruzeiro do Sul University, São Paulo, Brazil; 2 Department of Physiology and Biophysics, Institute of Biomedical Sciences, University of São Paulo, São Paulo, Brazil; 3 Department of Cell Biology and Development, Institute of Biomedical Sciences, University of São Paulo, São Paulo, Brazil; 4 Center of Physical Education and Sports, Federal University of Espirito Santo, Espirito Santo, Brazil; 5 Department of Medicine, Uniformed Services University of Health Sciences, Bethesda, Maryland, United States of America; National Institute of Agronomic Research, France

## Abstract

In this study, we investigated the effect of glutamine (Gln) supplementation on the signaling pathways regulating protein synthesis and protein degradation in the skeletal muscle of rats with streptozotocin (STZ)-induced diabetes. The expression levels of key regulatory proteins in the synthetic pathways (Akt, mTOR, GSK3 and 4E-BP1) and the degradation pathways (MuRF-1 and MAFbx) were determined using real-time PCR and Western blotting in four groups of male Wistar rats; 1) control, non-supplemented with glutamine; 2) control, supplemented with glutamine; 3) diabetic, non-supplemented with glutamine; and 4) diabetic, supplemented with glutamine. Diabetes was induced by the intravenous injection of 65 mg/kg bw STZ in citrate buffer (pH 4.2); the non-diabetic controls received only citrate buffer. After 48 hours, diabetes was confirmed in the STZ-treated animals by the determination of blood glucose levels above 200 mg/dL. Starting on that day, a solution of 1 g/kg bw Gln in phosphate buffered saline (PBS) was administered daily via gavage for 15 days to groups 2 and 4. Groups 1 and 3 received only PBS for the same duration. The rats were euthanized, and the soleus muscles were removed and homogenized in extraction buffer for the subsequent measurement of protein and mRNA levels. The results demonstrated a significant decrease in the muscle Gln content in the diabetic rats, and this level increased toward the control value in the diabetic rats receiving Gln. In addition, the diabetic rats exhibited a reduced mRNA expression of regulatory proteins in the protein synthesis pathway and increased expression of those associated with protein degradation. A reduction in the skeletal muscle mass in the diabetic rats was observed and was alleviated partially with Gln supplementation. The data suggest that glutamine supplementation is potentially useful for slowing the progression of muscle atrophy in patients with diabetes.

## Introduction

The maintenance of normal skeletal muscle mass and size is required for locomotion, heat production and the control of intermediary metabolism [1] and is dependent upon many factors, including the functioning of efferent motor innervation [2] and an adequate supply of glucose, fatty acids, and trophic hormones [3]. Diabetes mellitus, a disease affecting a quarter of a billion people worldwide [4], can cause skeletal muscle damage and atrophy via diabetic neuropathy and by the more direct effects of high glucose and low insulin [3] on muscle cell metabolism. Indeed, insulin is a major factor in the maintenance of the skeletal muscle protein mass [2], [5].

Muscle wasting in diabetes is ultimately the result of damage to the intracellular signaling pathways that are involved in maintaining the balance between protein degradation and new protein synthesis [5], which depends on both the phosphorylation and *de novo* expression of specific regulatory proteins. Specifically, the skeletal muscle mass is ultimately controlled by the signaling pathways leading to protein synthesis, especially the IGF-1/PI3K/Akt pathway and alternatively, pathways leading to degradation, such as MuRF-1- or MAFbx-dependent pathway intermediates [2], [6]. Depending on the physiologic state of the muscle cell, Akt transduces signals that lead primarily to increased protein synthesis (by activating a number of specific downstream proteins, including mTOR, p70s6k, 4E-BP1 and GSK3) or to decreased degradation (using pathways dependent upon MuRF-1 and MAFbx). Ultimately, the disruption of either pathway results in the increase in the overall muscle mass. In the protein synthetic pathway, Akt activation leads to the formation of a signaling complex termed TORC1, an important component of mTOR [7]. The activation of mTOR, in turn, induces the phosphorylation of p70S6K (activates the ribosomal subunit required for muscle protein translation) [8] and the translational repressor 4E-BP1, which is inactivated following phosphate addition [9].

The degradation pathway utilizes the ubiquitin ligases atrogin-1 (MAFbx) and MuRF1, and the increased expression of these enzymes is considered among the most reliable markers of muscle atrophy and wasting [10], [11]. The up-regulation of ubiquitin ligase, however, is inhibited by Akt via a mechanism involving members of the FOXO family of transcription factors [12], [13]. Specifically, Akt phosphorylates FOXOs (pFOXO), thereby promoting their migration from the nucleus to the cytosol. The reduction in the activity of the Akt pathway, as reported for several muscle atrophy models, causes a decrease in the cytosolic pFOXO and an increase in the nuclear FOXO protein that allows the up-regulation of atrogin-1/MAFbx and MuRF-1 and an increase in muscle atrophy [14].

Skeletal muscle overloading is a key activator of PI3K/Akt, leading eventually to muscle fiber hypertrophy [15]. TSC2, one of the proteins regulated by Akt kinase, is a constitutively active inhibitor of mTOR activity, and its activity is suppressed by the phosphorylation of specific amino acid residues. The result is an increase in protein translation and ultimately in muscle mass. Recently, it has been shown that the non-essential amino acid glutamine (Gln) may have a positive influence on the protein synthesis downstream of mTOR activation in a HeLa cell model [16]. It has been suggested that the mTOR activation in these cells is dependent on the uptake and subsequent rapid efflux of glutamine in the presence of essential amino acids [16]. Glutamine, at a concentration 0.5–0.8 mM in the blood and approximately 20 mM in the skeletal muscle, is the most abundant amino acid in the body [17], [18]. However, the precise role glutamine plays in regulating the skeletal muscle mass is not fully understood. A recent review described the ability of glutamine to regulate protein metabolism; however, the mechanism involved was not addressed [19].

Glutamine is a readily tolerated, naturally occurring substance, and the potential use of this amino acid for the treatment of chronic diseases is under investigation. In this study, we evaluated the possible beneficial effects of oral glutamine supplements on skeletal muscle morphology, glutamine/glutamate content, protein synthesis and degradation, determined by changes in the levels of mRNA expression and the activation of signaling molecules involved in protein synthesis (Akt, mTOR, GSK3 and 4E-BP1) and degradation (MuRF-1 and MAFbx).

## Materials and Methods

### Animals

Male Wistar rats were obtained from and housed at the Department of Physiology and Biophysics, Institute of Biomedical Sciences, University of São Paulo. The animals were maintained at 23±2°C under a cycle of 12-h light and 12-h darkness. The animals had free access to food (Nuvilab CR1, Nuvital Nutrientes Ltd., Curitiba, PR) and water. The animals were euthanized in a fed state. The experimental procedures were performed in strict accordance with the recommendations of the Guide for the Care and Use of Laboratory Animals, and the Ethical Committee of the Institute of Biomedical Sciences, University of São Paulo (Permit Number: 10/2008) approved this study. The animals were divided into four groups; 1) control, non-supplemented with glutamine; 2) control, supplemented with glutamine; 3) diabetic, non-supplemented with glutamine; and 4) diabetic, supplemented with glutamine.

### Induction of diabetes

The diabetic state was induced by a single intravenous injection of 65 mg/kg bw streptozotocin (STZ) dissolved in citrate buffer (pH 4.2). The control rats received an identical volume of buffer. After 48 hours, a diabetic state was confirmed by blood glucose levels above 200 mg/dL.

### Oral L-glutamine supplementation

A solution of glutamine (Gln), freshly prepared and dissolved in PBS, was administered by gavage once a day for 15 days (supplemented and diabetic-supplemented animals). The daily dose of glutamine (1 g/kg bw) was the same as previously described [20]. The non-supplemented rats (control and diabetic) received PBS alone. After 15 d of treatment, the plasma and muscle glutamine contents were determined using the method described by Windmueller and Spaeth [21].

### Effect of glutamine supplementation on the expression of signaling elements in protein synthetic and protein degradation pathways in skeletal muscle

After 15 d of supplementation with glutamine, the soleus muscles were removed and homogenized in extraction buffer (100 mM Trizma, pH 7.5; 10 mM EDTA; 100 mM NaF; 10 mM sodium pyrophosphate; 10 mM sodium orthovanadate; 2 mM phenylmethanesulfonyl fluoride; and 0.01 mg/mL aprotinin) at 4°C for 30 sec. After homogenization, Triton X-100 was added to a final concentration of 1%, the *s*amples were incubated for 30 min at 4°C and were centrifuged at 13,000× *g* for 20 min at 4°C. The total protein content was determined using bovine serum albumin as the standard [22].

Equal amounts of protein from each sample (75 µg) were diluted in Laemmli buffer containing dithiothreitol (DTT) (1 M) and were submitted to electrophoresis on polyacrylamide gels. The proteins were transblotted onto nitrocellulose membranes at 120 V for 1 h. The appearance of non-specific bands was blocked by pre-incubation of the membranes in basal solution (10 mM Trizma, pH 7.5; 150 mM NaCl; and 0.05% Tween 20) containing 5% skim milk at room temperature for 2 h. The membranes were washed 3 times (10 min each) in basal solution and were incubated with the following antibodies diluted in basal solution containing 3% skim milk, at room temperature for 3 h: Akt (1∶1,000 dilution), phosphor Akt (pAkt) (1∶1,000 dilution, Ser473), mTOR (1∶500 dilution), 4E-BP1 (1∶1,000 dilution), MuRF-1 (1∶500 dilution) and MAFbx (1∶500 dilution). The membranes were washed again (3×10 min) and incubated with the corresponding secondary antibody (1∶5,000) conjugated to horseradish peroxidase in basal solution, containing 1% skim milk, at room temperature for 1 h. Following a final wash, the membranes were incubated with the substrate for peroxidase and the chemiluminescence enhancer solution (ECL Western Blotting System Kit, GE Health Care, Little Chalfont, Buckinghamshire, England) for 1 min and were exposed immediately to X-ray film. The films were processed, and the band intensities were quantified by optical densitometry using the ImageJ 1.37 software (Wayne Rasband, NIH, USA; http://rsb.info.nih.gov/ij/). The band densities were normalized to an invariant band in membranes stained with Ponceau S.

### Real-Time PCR

The mRNA expression of the selected genes was evaluated by real-time PCR [23] using the ROTOR GENE 3000 apparatus (Corbett Research, Mortlake, Australia). The total RNA was obtained from 50 to 100 mg of the soleus muscle using Trizol reagent™ (Invitrogen Life Technologies, Rockville, MD, USA). Briefly, the soleus muscle was lysed using 1 mL Trizol reagent and after 5 min incubation at room temperature, 200 µL chloroform were added to the tubes, which were and centrifuged at 12,000× *g*. The aqueous phase was transferred to a separate tube, and the RNA was pelleted by centrifugation (12,000× *g*) with isopropyl alcohol. The RNA pellets were washed using 75% ethanol by centrifugation at 7,500× *g* for 5 min and were air-dried. The RNA pellets were eluted in RNase-free water and treated with DNAse I. Subsequently, the RNA was stored at −70°C until the reverse transcription procedure was performed. The RNA was quantified by measuring the absorbance at 260 nm. The purity of the RNA preparations was assessed by evaluating the 260/280 nm ratio and on a 1% agarose gel stained with ethidium bromide at 5 µg/mL.

The cDNA probes were synthesized using 4 µg of the total RNA and a mixture containing the following: 146 ng random primers, 200 U reverse transcriptase (Invitrogen Life Technologies, Rockville, MD, USA), 5× reaction buffer (50 mM Tris–HCl, pH 8.0; 75 mM KCl; 3 mM MgCl_2_), 5 mM DTT, and 500 µM dNTP in a final volume of 20 µL. The reaction was incubated for 2 min at 25°C, assembling the oligonucleotides and the RNA hybridization, followed by heating at 42°C for 50 min. The cDNA was stored at −20°C prior to the real-time PCR assay. For the real-time PCR reaction, 1 µg of cDNA in a final volume of 25 µL was used. The reaction mixture contained 100 µM dNTPs, 10× reaction buffer (10 mM Tris–HCl, 50 mM KCl, 2 mM MgCl_2_), 1 U Taq DNA polymerase (Invitrogen Life Technologies, Rockville, MD, USA), 0.1 µM of each primer (sense and antisense), and SYBR GREEN (diluted 1∶1,000) (Invitrogen Life Technologies, Rockville, MD, USA) was used as a fluorescent dye. The primer sequences were designed using the information contained in the Gene Bank of the National Center for Biotechnology Information (NCBI). The sense and anti-sense sequences and the annealing temperatures for Akt, 4E-BP1, GSK3, mTOR, MuRF-1, MAFbx, YWHAZ and B2M are shown in Table 1.

**Table 1:** Sequences of the primers, and annealing temperatures for the Real Time PCR of the genes studied.

*Gene*	*Primer*	*Annealing temperature (°C)*
Akt	Sense: GCCACAGGTCGCTACTATG	55.3
	Anti-sense: GCAGGACACGGTTCTCAG	
4E-BP1	Sense: CCTGATGGAGTGTCGGAAC	55.0
	Anti-sense: GAGGCTCATCGCTGGTAG	
GSK	Sense: CCACTCAAGAACTGTCAAG	50.5
	Anti-sense: CACGGTCTCCAGCATTAG	
mTOR	Sense: CAGGACGAGCGAGTGAT	55.4
	Anti-sense: CGAGTTGGTGGACAGAGG	
MuRF-1	Sense: CTATGGAGAACCTGGAGA	51.7
	Anti-sense: CCTGGAAGATGTCGTTGG	
MAF-bx	Sense: TGCTTACAACTGAACATC	45.6
	Anti-sense: TACATCTTCTTCCAATCC	

The quantification of gene expression was performed using the qBase software, as described previously [24]. B2M and YWHAZ were used as the internal controls using the geNorm application [25].

### Histological analysis

A cryostat was used to cut the muscle sections (10-µm thick) from the mid-belly region of the medial portion of the soleus muscle. The sections were stained using hematoxylin and eosin (HE) for the examination of the fiber cross-sectional area (CSA). The sections were photographed using an upright microscope equipped with a camera (Nikon DXM 1200, Japan). The digitized images were analyzed using the Image Pro Plus Software (Media Cybernetics, SilverSpring, MD) in a blinded manner. The mean fiber CSA was determined by measuring the circumference of 100 adjacent fibers from the center of each cross-section, totaling 600 fibers per muscle tissue.

### Statistical analysis

The data were analyzed using two-way analysis of variance (ANOVA) and the Bonferroni *post-hoc* test. The results were considered statistically significant at *P*<0.05. The GraphPad Prism 5 software (Graph Pad Software, Inc., San Diego, CA, USA) was used for the statistical analysis. For the histological analysis, the muscle cross-sectional area data were analyzed using the Anderson–Darling Normality Test. The muscle fiber CSA was not normally distributed; therefore, differences were considered significant when no overlap existed between the 95% confidence interval of the mean (95% CI).

## Results

**Figure 1:**
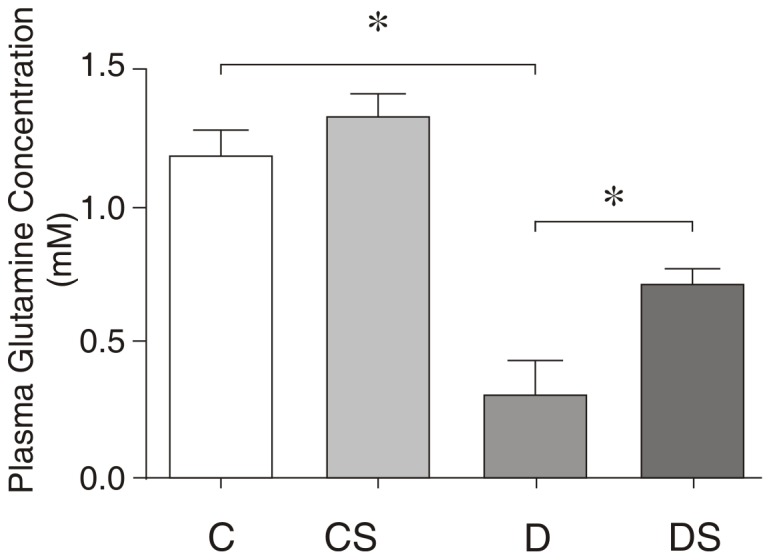
Plasma glutamine concentration (mM). The results are expressed as the means ± SEM. The values represent 6 animals/group. * *p*<0.05, as indicated by ANOVA and Bonferroni post-hoc test. C = control rats; CS = control rats supplemented with glutamine; D = diabetic rats; DS = diabetic rats supplemented with glutamine.

**Figure 2:**
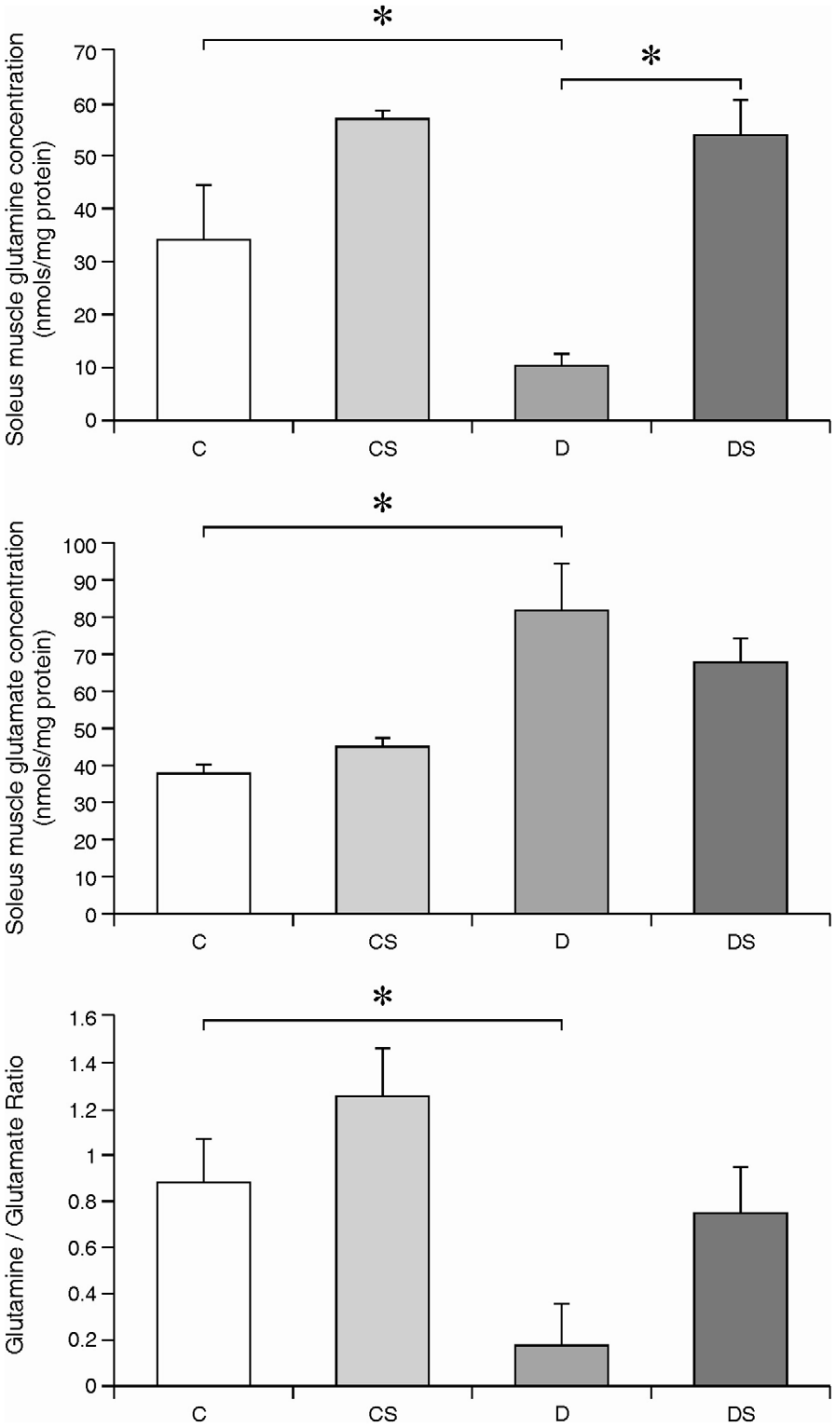
Soleus muscle glutamine concentration (nmols/mg protein) (A); soleus muscle glutamate concentration (nmols/mg protein) (B); glutamine/glutamate ratio (C). The results are expressed as the means ± SEM. The values represent 6 animals/group. * *p*<0.05, as indicated by ANOVA and the Bonferroni post-hoc test. C = control rats; CS = control rats supplemented with glutamine; D = diabetic rats; DS = diabetic rats supplemented with glutamine.

The plasma glutamine levels exhibited a marked decrease (∼80%) in the diabetic animals compared to the controls (Figure 1). In contrast, the plasma glutamate levels did not differ significantly between the two groups (data not shown). Likewise, the glutamine content of the soleus muscle was reduced to 30% of the non-diabetic control value in the STZ-diabetic animals, whereas the glutamate content was increased approximately 2-fold in the diabetic samples (Figure 2). Consequently, the glutamine/glutamate ratio of the muscle was decreased by 82% with the induction of the diabetic state (Figure 2).

In both the plasma (Figure 1) and soleus muscle (Figure 2), the glutamine supplementation of the diabetic animals (group DS) resulted in a significantly higher glutamine content compared with the non-supplemented diabetic controls (group D). The fold-increase in plasma Gln was 3.2-fold in the Gln-supplemented diabetic group (D vs. DS); however, exogenous Gln did not significantly alter the plasma Gln concentration in the non-diabetic animals (C vs. S). The glutamine/glutamate ratio in the soleus muscle was also elevated in the Gln-supplemented diabetic group compared with the non-supplemented diabetic group (Figure 2C).

**Figure 3:**
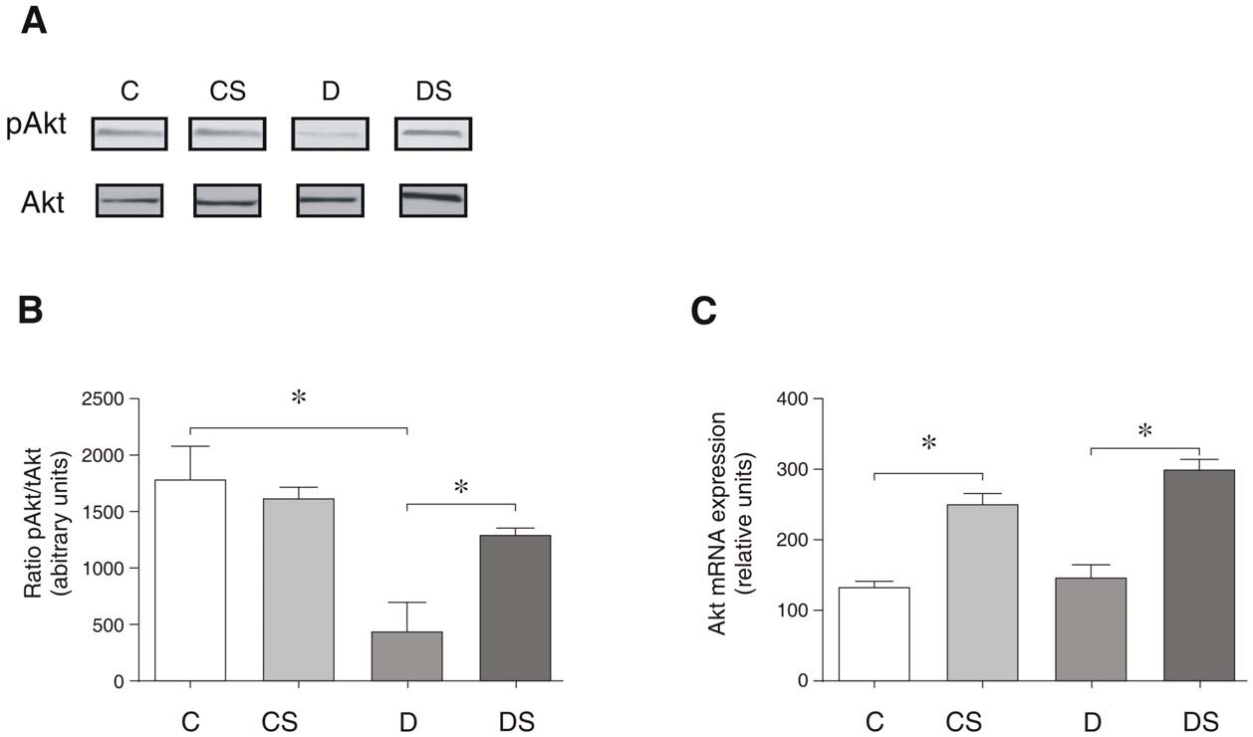
Representative Western blots for phosphorylated and total Akt (A); pAkt/tAKT ratio (B); Akt mRNA expression (C). The results are expressed as the means ± SEM. The values represent 6 animals/group. * *p*<0.05, as indicated by ANOVA and the Bonferroni post-hoc test. C = control rats; CS = control rats supplemented with glutamine; D = diabetic rats; DS = diabetic rats supplemented with glutamine.

Figure 3 demonstrates the effect of exogenous glutamine on the phosphorylation and gene expression of Akt. The diabetic animals exhibited markedly reduced levels of phosphorylated Akt (Figures 3A and B), with the ratio of pAkt to Akt decreasing 77% in these animals compared to the controls (Figure 3B). In contrast, the induction of the diabetic state had no effect on the mRNA levels of Akt (Figure 3C; C vs. D). The Gln supplementation did not change the Akt phosphorylation in the non-diabetic animals (C vs. D) but increased the Akt phosphorylation significantly (approximately 3-fold) in the diabetic animals (D vs. DS) (Figure 3B). Moreover, the Akt mRNA transcript levels were increased approximately 2-fold by the Gln supplementation in both the diabetic and non-diabetic animals (Figure 3C).

**Figure 4:**
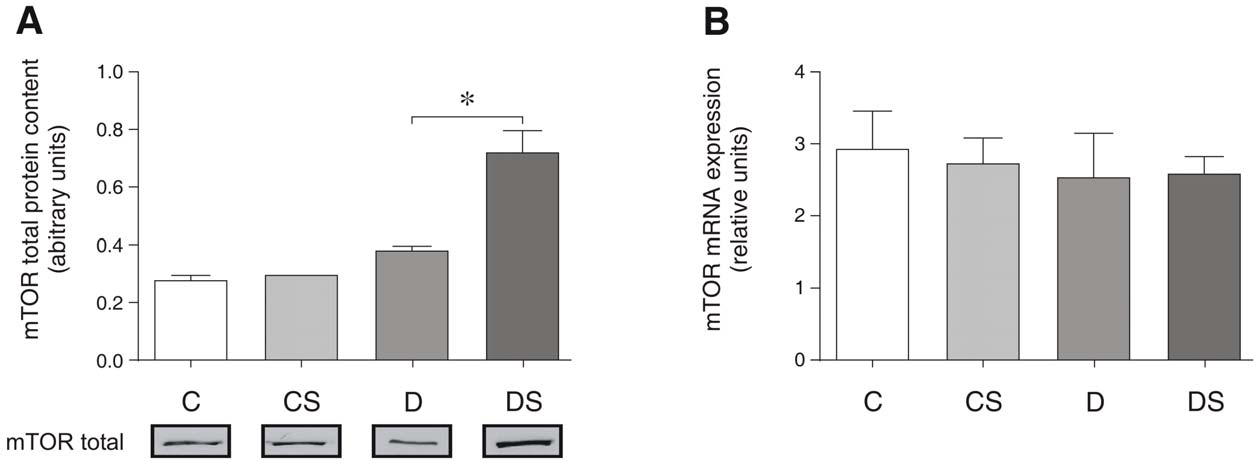
Representative Western blots for total mTOR protein content (A); mTOR mRNA expression (B). The results are expressed as the means ± SEM. The values represent 6 animals/group. * *p*<0.05, as indicated by ANOVA and the Bonferroni post-hoc test. C = control rats; CS = control rats supplemented with glutamine; D = diabetic rats; DS = diabetic rats supplemented with glutamine.

Unlike Akt, the diabetic state did not alter either the mTOR protein content (Figure 4A) or the gene expression significantly (Figure 4B). However, although the Gln supplementation failed to alter the mTOR protein levels in the non-diabetic animals, an almost 2-fold increase in the protein was observed in the diabetic animals (Figure 4A). The Gln effect was not observed at the mRNA level (Figure 4B). The induction of diabetes also failed to alter either the activation protein or gene expression of GSK3 (data not shown).

**Figure 5:**
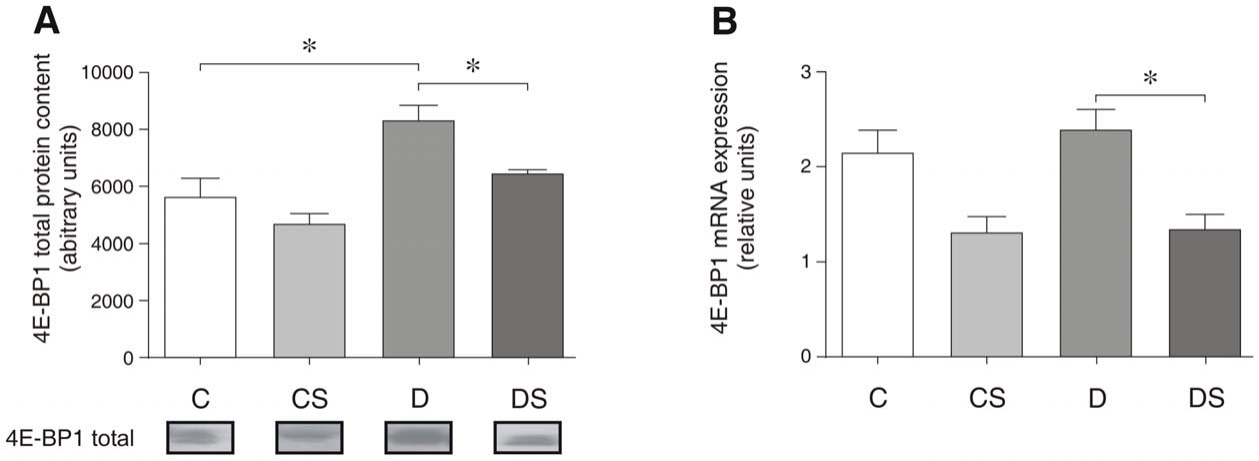
Representative Western blots for total 4E-BP1 protein content (A), 4E-BP1 mRNA expression (B). The results are expressed as the means ± SEM. The values represent 6 animals/group. * *p*<0.05, as indicated by ANOVA and the Bonferroni post-hoc test. C = control rats; CS = control rats supplemented with glutamine; D = diabetic rats; DS = diabetic rats supplemented with glutamine.

Similar to Akt, the 4E-BP1 mRNA expression in the soleus muscle was not altered significantly with the induction of diabetes (Figure 5B; C vs. D). However, the 4E-BP1 protein content was elevated significantly (48%) in the diabetic rats compared with the non-diabetic animals (Figure 5A). This increase in the 4E-BP1 protein content and mRNA expression levels in the diabetic animals was decreased significantly by the Gln supplementation (Figure 5B).

**Figure 6:**
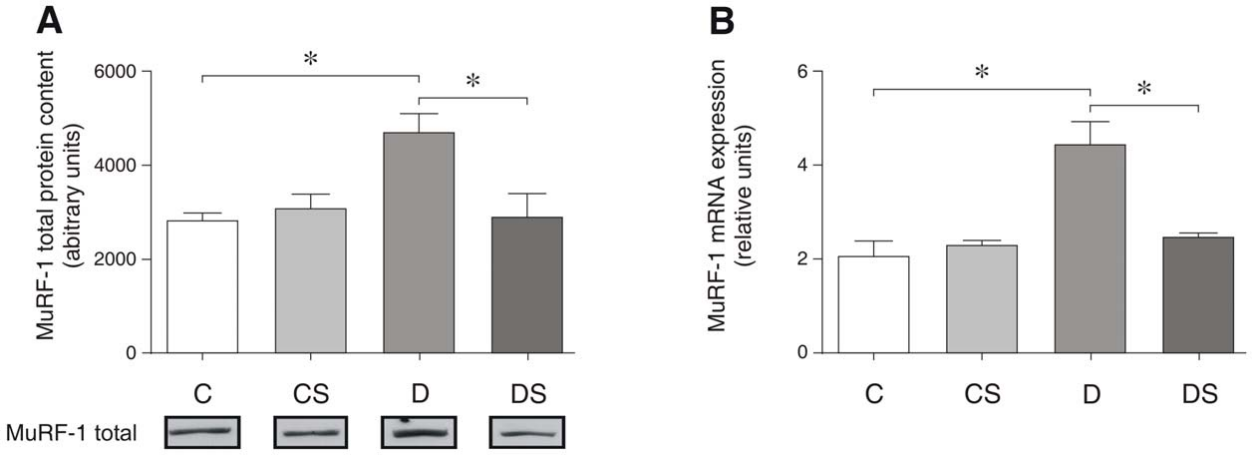
Representative Western blots for total MuRF-1 protein content (A); MuRF-1 mRNA expression (B). The results are expressed as the means ± SEM. The values represent 6 animals/group. * *p*<0.05, as indicated by ANOVA and the Bonferroni post-hoc test. C = control rats; CS = control rats supplemented with glutamine; D = diabetic rats; DS = diabetic rats supplemented with glutamine.

**Figure 7:**
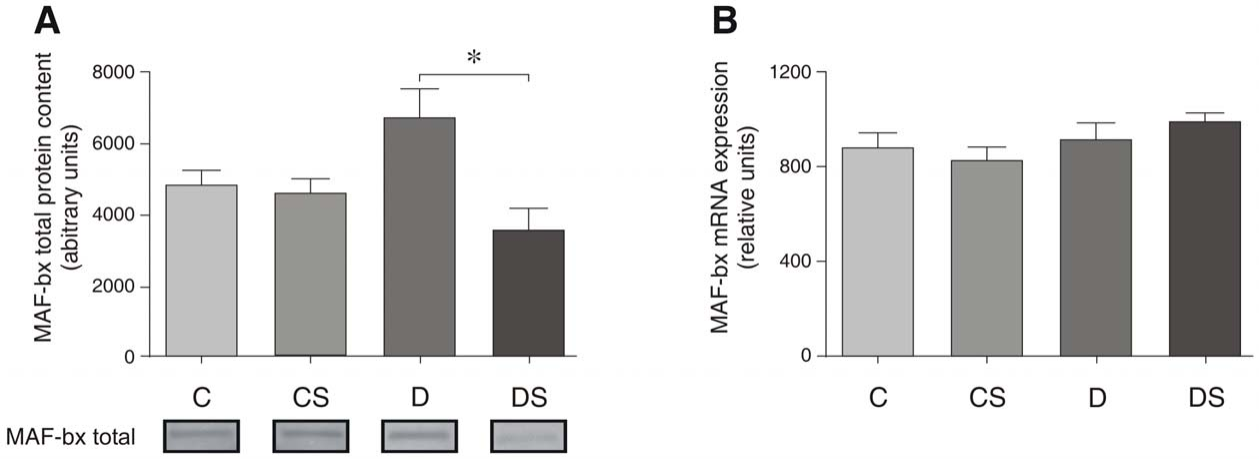
Representative Western blots for total MAFbx protein content (A); MAFbx mRNA expression (B). The results are expressed as the means ± SEM. The values represent 6 animals/group. * *p*<0.05 as indicated by ANOVA and the Bonferroni post-hoc test. C = control rats; CS = control rats supplemented with glutamine; D = diabetic rats; DS = diabetic rats supplemented with glutamine.

**Figure 8:**
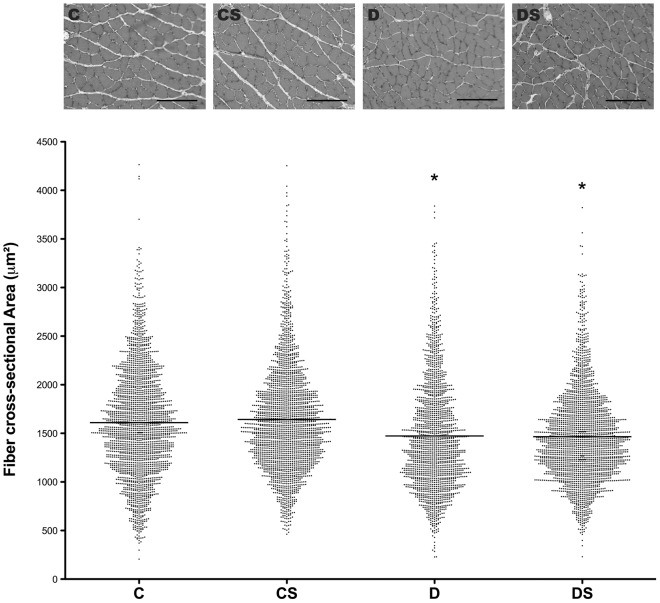
Soleus cross-sectional area (µm^2^) analysis. The results are expressed as the means ± SEM. The values represent 6 animals/group. * *p*<0.05, as indicated by the Anderson–Darling Normality Test. C = control rats; CS = control rats supplemented with glutamine; D = diabetic rats; DS = diabetic rats supplemented with glutamine.

Similar to mTOR and 4E-BP1, the protein degradation-associated genes MuRF-1 and MAF-bx were altered by the induction of diabetes, demonstrating significant increases at the protein or mRNA levels, or both (Figures 6 and 7). Similarly to its effect on 4E-BP1, Gln significantly reduced the mRNA levels of both MuRF-1 and MAF-bx. However, Gln only affected MAF-bx at the post-translational level, reducing the ubiquitin-related protein by approximately 50% without affecting mRNA (Figures 7A and 7B). The histological analysis of the soleus muscle demonstrated a small but significant difference in the fiber-cross sectional area between the control (1611±12 µm^2^) and the diabetic animals (1473±13 µm^2^), reflecting the loss of the myofibrils with the induction of diabetes (Figure 8). However, the 15-day Gln-treatment did not affect fiber cross-sectional area in either the non-diabetic (C vs. CS) or diabetic animals (D vs. DS) (1611±12 vs. 1643±11 and 1473±13 vs. 1466±10, respectively).

## Discussion

Our study demonstrates a number of significant differences in glutamine regulation and in the protein-synthetic and protein-degradative pathways in the skeletal muscle of the STZ-diabetic rats compared with the non-diabetic rats. We also demonstrate that the supplementation with exogenous glutamine reverses a number of these changes, emphasizing the importance of this amino acid in the etiology of diabetes and suggesting a potential role for Gln supplementation in suppressing/reversing the muscle loss associated with this disease.

A decrease in amino acid concentrations in the plasma has been reported previously for a variety of catabolic diseases, including diabetes [5], [26], [27] in which a reduction in the amino acid glutamine was observed in the plasma and skeletal muscle [5]. The underlying cause of the loss of skeletal-muscle Gln has been linked to the metabolic sequelae of increased liver gluconeogenesis and hyperglycemia [28].

In this study, we observed a significant reduction in plasma glutamine concentrations in the diabetic model compared with the non-diabetic animals, which is consistent with previous studies [5]. We also demonstrated that the glutamine supplementation markedly increased the plasma concentration of this amino acid in the diabetic rats but not in the non-diabetic rats. Although the Gln content of the skeletal muscle is lowered in diabetes, the glutamate content is increased, likely because of the increased activity of phosphate-dependent glutaminase, resulting in decreased Gln but increased glutamate as a product of glutamine deamination. The glutamine supplementation of the STZ-diabetic rats partially compensated for the Gln loss in the plasma (Figure 1) and restored the Gln content of the soleus to the non-diabetic control levels (Figure 2). Therefore, it is reasonable to assume that the oral administration of supplemental Gln resulted in sufficient plasma and intramuscular concentrations of the amino acid to alter the activity and/or expression of the signaling molecules in the direction of increased protein synthesis and decreased catabolism.

Akt is crucial to the potential role of Gln in attenuating diabetic muscle wasting, and the post-translational activity (i.e., phosphorylation) and in this study, the gene expression of Akt increased significantly following Gln supplementation. Previous investigations have demonstrated atrophic changes in skeletal muscle related to the reduced activity of Akt [1], [29] under conditions of mild insulinemia [3]. This study confirms the decreased activity of Akt activation in the diabetic state and demonstrates that diabetes affects neither the total protein content (data not shown) nor the mRNA expression of Akt in skeletal muscles. Nonetheless, because Gln supplementation increases Akt mRNA (Figure 3), Gln could have a potentially beneficial effect on diabetic skeletal muscles by increasing de novo Akt expression and synthesis.

Because Akt plays a key role in protein synthesis and protein degradation pathways as an activator and inhibitor, respectively, it is expected that following Gln supplementation, the restoration of the Akt activity and expression levels found in the control will prevent muscle wasting via the protective effects on *both* muscle anabolism and catabolism. Supporting a role for Akt/Gln in suppressing muscle breakdown, we demonstrate that both MuRF-1 and MAF-bx protein levels were decreased significantly in the diabetic rats receiving Gln (Figures 6 and 7).

In addition to insulin, several amino acids, leucine in particular, regulate protein synthesis [30] through the activation of PI3K and Akt. In one report, leucine supplementation in septic animals did not modulate the expression of Akt [31]. However, another study demonstrated that leucine treatment increased Akt phosphorylation in control mice treated with 1.5 mU/g of human insulin [32]. Leucine was also shown to ameliorate the increase in MAFbx and MuRF-1 caused by muscle disuse [33]. We demonstrated that in a diabetic rat model, treatment with glutamine (like leucine in earlier reports) results in Akt activation in skeletal muscle and induces increased Akt mRNA expression. These Gln-induced changes did not require co-treatment with insulin. In addition, consistent with the leucine effect on muscle disuse [33], the Gln supplementation in the diabetic rats decreased the synthesis of the muscle-specific ubiquitin ligases MAFbx and MuRF-1, likely downstream of the phosphorylation of FOXO by Akt [12], [34].

The similarities between the leucine- and glutamine-mediated regulation of protein synthetic/degradative pathways may be in part because both amino acids use the same heterodimeric SLC7A5/SLC3A2 bidirectional transporter for influx (leucine) or efflux (glutamine) from the cell [16], [35]. This transporter uses intracellular glutamine as an efflux substrate to take up extracellular leucine and to activate mTORC1. Indeed, glutamine is an essential and rate-limiting factor for the activation of mTORC1 by essential amino acids (such as leucine) and growth factors, initiating the process of protein translation (described in the Introduction). The intracellular glutamine required for the activation of the bidirectional transporter enters the cell through the high-affinity L-glutamine transporter SLC7A5 [16].

Of the signaling proteins examined in this study, the activity and mRNA expression of only GSK3 was unchanged in the Gln-supplemented rats compared with the control rats. GSK3 is considered a key factor in the development of insulin resistance and type II diabetes [36], and GSK3 inhibition improves skeletal muscle insulin resistance in type II diabetic animals [37]. Our demonstration of the effect of Gln is consistent with other reports [38] showing that in the diabetic state, insulin and vanadium treatments do not affect the GSK3 activity in the skeletal muscle.

The effects of insulin on muscle and other tissues may occur directly through the activation of Akt and mTOR, or the effects might be conveyed indirectly through changes in the intracellular amino acid levels. Although not yet fully defined [39], evidence suggests that the mechanism of insulin action may occur through the activation of the class III PI3 kinase (hVps34) [40]. Therefore, the low plasma insulin levels observed in the STZ-induced diabetes is expected to lower the activation of Akt, as demonstrated in this study. However, our data also indicate that the diabetic state did not influence the expression of the mTOR complex (Figure 4). Remarkably, in the diabetic rats, Gln supplementation increased the mTOR protein content to double that of the control (Figure 4A). In contrast, the non-diabetic rats did not exhibit increased levels of the mTOR protein following Gln supplementation. These results suggest that the diabetic state is required for this particular Gln-mediated event in the protein synthetic pathway to occur.

The diabetic state was also required for the effect of Gln supplementation on the 4E-BP1, MuRF-1, and MAF-bx protein content. It is plausible that the synthesis of these four proteins, including mTOR (two of which are in the protein-synthetic pathway and two are involved in protein degradation) is in some way dependent on an insulin- or IGF-1-mediated alteration of a common regulatory protein. However, the possible dependence of these effects on insulin signal transduction is difficult to identify. Knockout animals for the insulin receptor (MIRKO) that received exogenous insulin exhibited decreased MAFbx (4-fold) and MuRF-1 (2.3-fold) mRNA expression in the gastrocnemius muscle, suggesting a signaling mechanism that largely bypasses the insulin receptor. Regardless of the level that the glutamine effect on the muscle is altered by diabetes, our results suggest that at the clinical level, Gln supplementation is essentially ‘targeted’ to diabetic individuals, producing little or no effect on muscle function in normal individuals.

The histological analysis in this study clearly showed the atrophic effect of STZ-diabetes in skeletal muscle; the cross-sectional muscle fiber area was reduced by 9% (Figure 8). However, in this study, Gln supplementation did not have a significant effect on muscle atrophy, in either the diabetic or non-diabetic animals. We speculate that the 15-day period of Gln supplementation, although sufficient to detect molecular changes in the muscle signaling pathways, was not sufficient to allow the beneficial effects of Gln to be translated into an increase in the muscle mass. In summary, this study identifies the specific molecular changes in the skeletal muscle signaling pathways in the STZ-diabetic rat, all of which are consistent with the eventual loss of muscle mass characteristic of this disease. Our finding that glutamine supplementation in the diabetic rats reversed most of the deleterious changes in the signaling molecules suggests that oral supplementation with Gln could prove an inexpensive and well-tolerated treatment for the progressive muscle wasting in diabetes.
