# Correction: Glutamine Supplementation Stimulates Protein-Synthetic and Inhibits Protein-Degradative Signaling Pathways in Skeletal Muscle of Diabetic Rats

**DOI:** 10.1371/annotation/a3cda985-fb83-49fb-9cb3-8dab0e355ddd

**Published:** 2013-06-03

**Authors:** Adriana C. Lambertucci, Rafael H. Lambertucci, Sandro M. Hirabara, Rui Curi, Anselmo S. Moriscot, Tatiana C. Alba-Loureiro, Lucas Guimarães-Ferreira, Adriana C. Levada-Pires, Diogo A. A. Vasconcelos, Donald F. Sellitti, Tania C. Pithon-Curi

After the publication of this manuscript we observed mistakes in Figures 3A, 4A, and 6A. The representative images related to pAkt (Figure 3A), mTOR total (Figure 4A), and MuRF-1 total (Figure 6A) have been revised. Please note the original raw blots are now provided with the revised Figures as part of this Correction.

In Figure 3A, pAkt panel, the C and CS bands had been duplicated.

In Figure 4A, the bands were re-arranged compared to the original blot.

In Figure 6A, the band for group D was incorrect.

The remaining Figures, results and conclusions are the same as originally reported in the article. The authors apologize for these errors and refer readers to the corrected Figures 3A, 4A, and 6A provided in this Correction.

Figure 3A: http://www.plosone.org/corrections/pone.0050390.g003a.cn.tif

Figure 3A blot: http://www.plosone.org/corrections/pone.0050390.g003a_blot.cn.tif

Figure 4A: http://www.plosone.org/corrections/pone.0050390.g004a.cn.tif

Figure 4A blot: http://www.plosone.org/corrections/pone.0050390.g004a_blot.cn.tif

Figure 6A: http://www.plosone.org/corrections/pone.0050390.g006a.cn.tif

Figure 6A blot: http://www.plosone.org/corrections/pone.0050390.g006a_blot.cn.tif

